# Trephining for Insanity

**Published:** 1890-05

**Authors:** 


### TREPHINING FOR INSANITY.

Brain surgery has taken a wonderful stride, even in the last five years, and the operation of trephining is now often performed and in quite a variety of diseases. One of its latest applications was in a case of general paralysis, which, when it starts, as a rule, goes on as relentlessly as fate. The patient was a man in whom the disease had made considerable progress, and death seemed not far away. He was trephined, and an opening made in his skull one and one-half inches long by three-quarters of an inch wide. This was made with a view of relieving the tension due to the pressure of fluid present in the brain ; also to arrest the irritative changes going on. The man was insane before the operation, but his mind cleared up after it, and at last reports was doing well. Not impossibly the time is coming when certain forms of insanity will be numbered among the surgical diseases.

